# Changes in Maxillary Incisor Inclination Before and After Orthodontic Treatment Across Vertical Skeletal Patterns

**DOI:** 10.3390/diagnostics15222933

**Published:** 2025-11-20

**Authors:** Samar Bou Assi, Antoine E. Hanna, Rita Pamela Khoury, Anthony T. Macari

**Affiliations:** 1 Division of Orthodontics and Dentofacial Orthopedics, Department of Dentofacial Medicine, Faculty of Medicine, American University of Beirut, P.O. Box 11-0236, Beirut 1107 2020, Lebanon; assisamar@hotmail.com (S.B.A.); ah111@aub.edu.lb (A.E.H.); rk225@aub.edu.lb (R.P.K.); 2 Department of Orthodontics, Faculty of Dental Medicine, Lebanese University, P.O. Box 11-0236, Beirut 1107 2020, Lebanon

**Keywords:** orthodontics, incisor, cephalometry, facial pattern, dental esthetics

## Abstract

**Objective**: To evaluate changes in maxillary incisor inclination before and after orthodontic treatment in adults with different vertical facial patterns (normodivergent, hypodivergent, hyperdivergent) and to assess the relationship of incisor inclination to facial and growth axes using cephalometric and photographic records. **Materials and Methods**: This retrospective study included 144 non-growing patients (96 females, 48 males) with available pre- and post-treatment lateral cephalograms and smiling profile photographs. Patients were classified into three groups based on mandibular plane angle (MP/SN): normodivergent (*n* = 66), hypodivergent (*n* = 35), and hyperdivergent (*n* = 43). Angular measurements assessed maxillary incisor inclination and growth/facial axes. Clinical crown angulation (CCA) was evaluated from profile photographs. Statistical analyses included paired *t*-tests, ANOVA with Bonferroni post hoc tests, and Pearson correlation. **Results**: No significant changes in maxillary incisor inclination were observed post-treatment in any of the groups. Significant skeletal changes were noted in the hypodivergent group, including increases in MP/SN (*p* = 0.011) and IMPA (*p* = 0.014). Intergroup comparison revealed significant differences in changes in Facial Axis/H (*p* = 0.020) and MP/SN (*p* = 0.025). Correlations between CCA and skeletal axes were more pronounced in normo- and hypodivergent groups, while hyperdivergent patients showed no significant associations. **Conclusions**: These findings suggest that the stability of maxillary incisor inclination reflects controlled torque mechanics during treatment. In normo- and hypodivergent patients, skeletal axes may help guide esthetic incisor positioning; however, in hyperdivergent patients, soft tissue and smile evaluation should play a greater role when determining final incisor inclination.

## 1. Introduction

With growing awareness of facial and smile esthetics, most individuals seeking orthodontic treatment aim to improve both dental alignment and smile attractiveness. Evaluating the inclination of maxillary anterior teeth is thus a routine step in orthodontic planning to ensure proper positioning within the basal bone and in harmony with facial features. Specifically, assessing the inclination and position of the maxillary incisors (MI) is essential for treatment planning, monitoring progress, and achieving esthetic outcomes [1,2].

Maxillary incisors inclination can be evaluated using various reference lines and diagnostic tools, including lateral cephalograms, dental casts, and smiling profile photographs [3,4,5,6]. Regardless of the method, orthodontists must assess incisor inclination at the start, monitor it throughout treatment, and ensure an optimal final position for esthetics and function.

Cephalometric analysis remains a standard method for assessing MI inclination, typically by measuring the angle between the incisor axis (from incisal tip to apex) and planes such as Sella-Nasion (SN), Frankfort Horizontal (FH), Palatal Plane (PP), A-Pogonion (A-Pog), N-A, N-Pog, a line perpendicular from Nasion through A-point, the maxillary occlusal plane, the bony orbit, and the interincisal angle [7,8,9,10]. These assessments must account for various influencing factors [7,8,9,10].

While much attention has been given to cephalometric incisor inclination, less is known about its relation to facial pattern, particularly facial axes. Ricketts’ analysis defines the facial axis (FA) as the angle between Ba-N and Pt-Gn lines, with a mean of 90° ± 3.5°, indicating mandibular growth direction [7,11]. Similarly, Downs’ growth axis (GA), formed by S-Gn and FH lines, reflects mandibular growth pattern, averaging 59.4° ± 3.8° [7,12]. Clinically, we have noted that ideally inclined maxillary incisors often appear nearly parallel to the facial axis.

The inclination of the maxillary incisors can also be assessed using facial profile photographs. One method uses forehead landmarks to evaluate the anteroposterior position of the maxillary central incisors in relation to the facial profile. It was found that 93% of individuals with harmonious profiles had their incisors positioned between the forehead’s FFA point and the glabella [13]. This technique helps assess the correlation between forehead prominence and incisor inclination. Another approach evaluated the esthetic impact of maxillary incisor inclination using smiling profile photographs. Angular measurements were made relative to the true horizontal, the tangent to the labial surface of the maxillary central incisor, and the line joining subnasale (Sn) to soft tissue pogonion (Pg′), to determine the most esthetic inclination and its correlation with facial features [14]. Similarly, another study examined the influence of labiolingual inclination of maxillary incisors on smile esthetics, using the same angular references—Sn/Pg′, the labial tangent, and true horizontal—to determine which inclinations were perceived as most esthetic in profile view [15]. These photographic assessments offer valuable insight into the esthetic evaluation of maxillary incisor inclination and its impact on facial and smile attractiveness.

While previous studies have described normative values for maxillary incisor inclination, fewer have examined how these values relate to vertical facial divergence and whether this relationship is maintained after orthodontic treatment. Understanding this relationship is clinically important because facial divergence influences growth direction, soft tissue balance, and esthetic smile display. Therefore, this study aimed to evaluate changes in maxillary incisor inclination before and after orthodontic treatment in adults with different vertical skeletal patterns and to assess how these inclinations relate to facial and growth axes.

The objectives of this study were: 1—To assess whether maxillary incisor inclination and facial/growth axes differ before and after treatment across three vertical skeletal patterns (normodivergent, hypodivergent, hyperdivergent) using lateral cephalograms and smiling profile photographs. 2—To compare pre- and post-treatment changes in maxillary incisor inclination relative to facial and growth axes on both lateral cephalograms and smiling profile photographs.

The null hypotheses were that no significant differences exist in maxillary incisor inclination before and after treatment among the vertical or sagittal groups, and that no changes occur in incisor inclination relative to facial and growth axes on either cephalometric or photographic records.

## 2. Materials and Methods

This retrospective comparative study was approved by the Institutional Review Board (IRB) at the American University of Beirut (OTO.AM.01, 10 June 2014). Consent to participate was deemed unnecessary.

Using an anticipated effect size (f2) of 0.02 (large) and a power level of 0.8 with three main predictors and a probability level of 0.05.

Patient records from the Department of Dentofacial Medicine at the American University of Beirut, Beirut, Lebanon, were selected based on the following inclusion criteria: Chronological age above 16 years for females and above 18 years for males, and availability of lateral cephalometric radiographs taken both before and after orthodontic treatment (after appliance removal), as well as smiling profile photographs taken at the same two time points. Patients with craniofacial anomalies and/or a history of orthognathic surgery were excluded. Patients were treated by multiple practitioners within the same academic orthodontic department; however, all cases followed standardized treatment protocols, including a uniform bracket prescription and finishing objectives, to ensure consistency in torque and treatment outcomes.

A total of 144 non-growing patients were included in the study (96 females, 48 males). Each had lateral cephalometric radiographs taken before and after orthodontic treatment in the same machine, acquired in natural head position at a standardized sagittal plane-to-film distance of 13 cm. All pre-treatment lateral cephalometric radiographs demonstrated cervical vertebral characteristics consistent with CVM Stage VI, indicating that skeletal growth had ceased and that the included individuals were in a non-growing stage at the time of treatment.

Patients were treated using individualized treatment plans that included both extraction and non-extraction protocols depending on clinical needs; however, treatment modality was not used as a grouping variable, as the study focused on changes in maxillary incisor inclination in relation to vertical divergence pattern rather than type of treatment. All patients were treated with fixed orthodontic appliances using 0.022″ × 0.028″ Roth prescription brackets.

The 288 lateral cephalograms (144 at T1 and 144 at T2) were digitized using Dolphin Orthodontic software (Version 11; Dolphin Imaging and Management Solutions, La Jolla, CA, USA).

**Figure 1:**
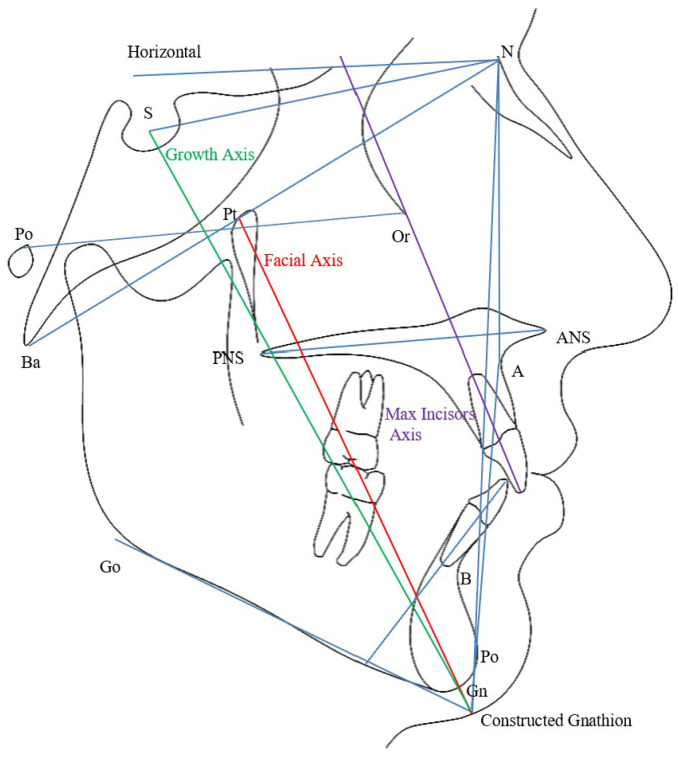
Lateral cephalometric tracing with landmarks and planes used in the study.

Various angular cephalometric measurements were performed on the digitized images to assess the inclination of the maxillary incisors relative to SN, PP, NA, NBa, and the true horizontal (H). Additionally, the inclinations of the facial and growth axes were measured with respect to NBa and true horizontal (H). Other variables recorded included the interincisal angle, IMPA, ANB, and MP/SN angles (Figure 1).

The sample was divided into three groups based on the vertical pattern (MP/SN): Normodivergent group (27° < MP/SN < 37°; *n* = 66); Hypodivergent group (MP/SN ≤ 27°; *n* = 35); Hyperdivergent group (MP/SN ≥ 37°; *n* = 43).

In addition, extraoral smiling profile photographs of the 144 patients were analyzed at two time points: baseline (T1) and at the end of treatment (T2). All profile photographs were taken with the patients positioned in their natural head position (NHP), defined as the upright, self-balanced posture that an individual naturally adopts in the absence of visual cues [16].

**Figure 2:**
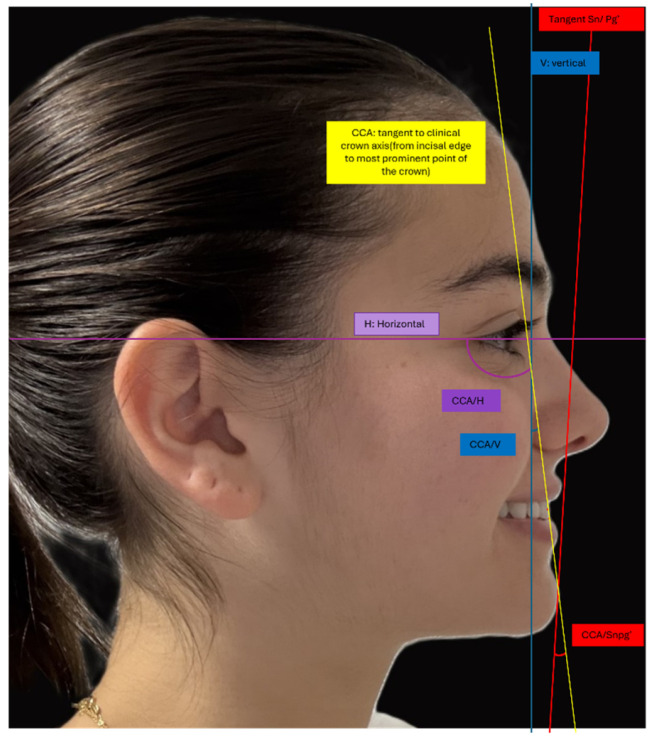
Smiling profile photograph with soft tissue landmarks, planes and angles used in the study.

Three angular measurements were assessed from the profile photographs (Figure 2):

1. **CCA-H**: The angle between the clinical crown axis (CCA) and the horizontal reference line (H). The CCA is defined as the line extending from the incisal edge of the maxillary central incisor to the most prominent point on the labial surface of the crown.
2. **CCA–Sn-Pg′**: The angle between the CCA and the line connecting subnasale (Sn) to soft tissue pogonion (Pg′). The subnasale (Sn) is the deepest point at the junction of the nose and upper lip, where the nasal septum meets the upper cutaneous lip in the midsagittal plane. Soft tissue pogonion (Pg′) is the most anterior point on the chin.
3. **CCA-V**: The angle between the CCA and the true vertical (V). The true vertical represents the gravitational vertical.

If the CCA is positioned anterior to the Sn–Pg′ line or the vertical line, the angle is considered positive; if posterior, it is considered negative.

## 3. Statistical Analysis

Initially, a frequency distribution was generated for all variables to check for any potential outliers and perform data cleaning. Interrater reliability was assessed using the intraclass correlation coefficient.

To assess differences between T1 and T2, paired sample *t*-tests were used.

When groups were stratified based on the vertical MP/SN, the one-way analysis of variance (ANOVA) was used to test differences in variables between these groups followed by the post hoc Bonferroni test. The chi-square test was used to gauge bivariate associations between malocclusion and vertical patterns. Post hoc results for the chi-square test were calculated by extracting the adjusted residuals. For all parameters, two-sided *p* values were reported. *p* value < 0.05 was considered statistically significant. All analyses were completed using IBM SPSS Statistics version 27 (IBM, released 2020, IBM SPSS Statistics for Windows, version 27.0, Armonk, New York, NY, USA).

Following data collection, a post hoc power analysis was conducted using a medium effect size of 0.15, α = 0.05, *n* = 144 with 4 key predictors: divergence, upper incisor inclination, facial axis and growth axis. The power obtained was 0.97.

Cephalometric and photographic tracings were performed by a single calibrated examiner. To assess measurement reliability, 10% of the records were retraced after a two-week interval by the same examiner (intra-examiner reliability) and independently by a second examiner (inter-examiner reliability).

## 4. Results

The intraclass correlation coefficients for both intra- and inter-examiner assessments were all greater than 0.90, indicating excellent measurement consistency.

A total of 66 participants were included in the normodivergent group (Normo), 35 in the hypodivergent group (Hypo), and 43 in the hyperdivergent group (Hyper). Each group was subdivided into T1 (initial) and T2 (follow-up) time points.

In the normodivergent group, no statistically significant differences were observed between T1 and T2 across all variables. Angular measurements related to maxillary incisor inclination (I/NA, I/PP, I/SN), sagittal skeletal relationship (ANB), facial and growth axis indicators, and dental parameters (MP/SN, IMPA, interincisal angle) remained stable over time (all *p* > 0.05) (Table 1).

In the hypodivergent group, a significant increase in mandibular plane angle (MP/SN) was observed from 23.86° ± 4.31° to 25.30° ± 5.08° (*p* = 0.011), as well as a significant increase in mandibular incisor inclination (IMPA) from 96.01° ± 7.71° to 98.97° ± 8.86° (*p* = 0.014). Other parameters, including maxillary incisor inclination and skeletal axes, did not show statistically significant changes (*p* > 0.05) (Table 1).

In the hyperdivergent group, no statistically significant differences were noted between T1 and T2 in maxillary incisor inclination (I/NA, I/PP, I/SN), facial/growth axis parameters, or mandibular plane angle (all *p* > 0.05), although a trend toward an increase in IMPA (*p* = 0.064) and decrease in interincisal angle (*p* = 0.074) was observed (Table 1).

Maxillary incisors’ clinical crown angulation (CCA) as measured in the profile smile, including CCA/TV, CCA/Sn′, and CCA/H, did not exhibit statistically significant changes between T1 and T2 in any of the three skeletal divergence groups. In the normodivergent group, all three measurements remained relatively unchanged (e.g., CCA/TV: −4.74° ± 9.12° at T1 vs. −4.62° ± 6.84° at T2, *p* = 0.902). Similarly, in both the hypodivergent and hyperdivergent groups, changes over time were minimal and did not reach statistical significance (all *p* > 0.05) (Table 2).

### 4.1. Comparison of Cephalometric Changes Among Skeletal Divergence Groups

To evaluate intergroup differences in cephalometric changes over time, delta (δ) was calculated (T2 − T1) for each variable, and then compared between different divergent groups.

Most variables showed no statistically significant differences between the normodivergent, hypodivergent, and hyperdivergent groups (*p* > 0.05) (Table 3). These included age change, maxillary incisor inclinations relative to NA, PP, SN, NBa, and H planes, as well as skeletal parameters such as ANB angle, growth and facial axes (relative to both NBa and H), and the interincisal angle.

However, two variables demonstrated statistically significant intergroup differences:

1. Facial axis to horizontal plane (Facial axis/H) showed a significant difference between groups (*p* = 0.020). Post hoc comparisons indicated a significant difference between the normodivergent and hyperdivergent groups (*p* = 0.019), with no significant differences between the normodivergent and hypodivergent (*p* = 1.000) or the hypodivergent and hyperdivergent groups (*p* = 0.172) (Table 3).
2. Mandibular plane angle relative to SN (MP/SN) also showed a significant difference among the groups (*p* = 0.025). The difference between hypodivergent and hyperdivergent groups was statistically significant (*p* = 0.021), whereas no significant differences were found between normodivergent and hypodivergent (*p* = 0.197) or normodivergent and hyperdivergent groups (*p* = 0.694) (Table 3).

### 4.2. Correlations Between Maxillary Incisors Inclination and the Facial and Growth Axes Before (T1) and After (T2) Orthodontic Treatment

At T1, significant positive correlations were observed between the maxillary incisor clinical crown axis (CCA) and the facial axis/NBa in both hypodivergent (r = 0.44, *p* < 0.001) and normodivergent (r = 0.37, *p* < 0.001) groups, indicating a moderate alignment between incisor inclination and this skeletal reference in subjects with lower and average vertical facial patterns. Similarly, CCA also correlated significantly with the facial axis/H in the hypodivergent (r = 0.44, *p* < 0.001) and normodivergent (r = 0.30, *p* < 0.05) groups. In contrast, no significant correlations were found in the hyperdivergent group at T1. As for the G-axis/NBa, a weak but significant correlation was found only in the normodivergent group at T1 (r = 0.25, *p* < 0.05). At T2, a notable shift was observed in the hypodivergent group, where CCA/V showed a significant negative correlation with G-axis/NBa (r = −0.34, *p* < 0.05), and CCA/SnPg′ demonstrated a strong negative correlation (r = −0.52, *p* < 0.001). Additional negative correlations at T2 were seen between CCA/SnPg′ and G-axis/H in both the hypodivergent (r = −0.34, *p* < 0.05) and normodivergent (r = −0.27, *p* < 0.05) groups. The hyperdivergent group consistently showed no significant correlations at either time point. These findings suggest that the relationship between incisor inclination and skeletal axes is more pronounced in hypodivergent and normodivergent individuals, and that this association weakens or becomes inconsistent in hyperdivergent cases (Table 4).

## 5. Discussion

The inclination of maxillary incisors plays a critical role in the smile esthetics, influencing both dental appeal and facial harmony. This study examined the effects of orthodontic treatment on maxillary incisor inclination in adults with normodivergent, hypodivergent, and hyperdivergent patterns. Results revealed that maxillary incisor inclination remained stable post-treatment across all groups, with no significant intra- or intergroup differences. These findings align with Hussels and Nanda [17], who noted that when initial incisor angulation is within normative limits, it tends to remain unchanged. This likely reflects clinicians’ preference to preserve esthetic norms during treatment planning.

While incisor inclination was maintained, notable skeletal changes occurred, especially in mandibular plane angle (MP/SN) and lower incisor inclination (IMPA) among hypodivergent patients. This suggests a compensatory dentoalveolar response in low-angle cases, consistent with observations by Bishara and Jakobsen [18] and supported by Assi et al., who reported that vertical patterns affect mandibular dynamics more than maxillary torque [19].

From an esthetic standpoint, Naini et al. determined that an 85° maxillary incisor inclination to the true horizontal was most attractive, while inclinations over 105° were rated poorly [20]. Albwardi et al. similarly found that labial inclinations beyond +15° reduced smile attractiveness [15]. Ghaleb et al. reported orthodontists to be particularly sensitive to changes in incisor torque, more so than laypersons or general dentists [14].

Smile esthetics is multifactorial. Cheng et al. and Palmares et al. emphasized the importance of overjet, gingival display, and facial proportions [21,22], while Flores-Mir et al. showed that laypeople’s perceptions are influenced by various dental and facial features [23].

Although soft tissue parameters such as CCA/TV, CCA/Sn′, and CCA/H remained unchanged in our sample, soft tissue plays a central role in smile design. Sarver and Ackerman argued that adult soft tissue profiles are relatively stable, and skeletal changes may not significantly alter facial esthetics [24]. Similarly, Cao et al. showed that excessive maxillary proclination negatively affects profile esthetics, despite minimal soft tissue adaptation [2].

In borderline Class III cases, incisor inclination control is vital. Burns et al. and Troy et al. found that retroclination often defines the feasibility of non-surgical options [25,26]. Kau et al. reported variable inclination outcomes across skeletal classes [27].

Growth and facial axes are cephalometric indicators of skeletal harmony. A forward-directed facial axis usually reflects a balanced Class I profile, while a vertically oriented growth axis suggests favorable horizontal development. Conversely, backward-rotated axes may reflect skeletal discrepancies, particularly in Class II or hyperdivergent patterns. Evaluating these axes aids in diagnosing jaw relationships and planning appropriate interventions, whether orthodontic or surgical. In hyperdivergent patients, characterized by clockwise growth and vertical excess, a significant difference in the Facial Axis/H relative to normodivergent individuals was observed, echoing findings by Schudy and Enlow on skeletal divergence and facial height [28,29]. Nevertheless, incisor inclination remained stable, underscoring the orthodontist’s emphasis on preserving facial esthetics by minimizing excessive compensation.

Minimal changes in interincisal angle were observed, consistent with Zierhut et al. who noted that adult orthodontic treatment tends to maintain existing anterior relationships when angulation is appropriate [30]. Chirivella et al. similarly emphasized the esthetic impact of incisor positioning across facial types [31].

Smile arc design further highlights the importance of incisor positioning. Sarver stressed its alignment with the lower lip, while Ackerman and Ackerman advocated for digital analysis to account for dynamic facial movements [32,33]. Optimal axial positioning supports occlusion, smile symmetry, lip support, and overall esthetics [16,34].

Our data showed significant correlations between maxillary incisor clinical crown axis (CCA) and skeletal reference planes, especially in hypodivergent and normodivergent adults. At T1, CCA was moderately correlated with the facial axis/NBa and facial axis/H in these groups, but not in hyperdivergent group. After treatment (T2), these associations weakened, with hypodivergent patients showing new negative correlations, notably between CCA/SnPg′ and G-axis/NBa. Hyperdivergent individuals displayed no significant correlations at either time point, suggesting a decoupled relationship between dental and skeletal parameters in this group.

### Clinical Implications, Limitations, and Future Directions

The findings of this study suggest that in normodivergent and hypodivergent patients, the inclination of the maxillary incisors can be guided reliably using cephalometric skeletal axes, as these patterns demonstrated a more stable relationship between dental and skeletal reference planes. Clinically, this may assist orthodontists in setting and maintaining an ideal torque and esthetic incisor position at the end of treatment. In contrast, hyperdivergent patients exhibited weaker skeletal and dental associations, indicating that soft tissue evaluation, smile dynamics, and individualized esthetic goals should play a greater role in final maxillary incisor positioning in this group.

A limitation of the present study is the variability in sample size across the divergence groups, which may influence the comparative power of intergroup analyses. Additionally, the retrospective design and the use of two-dimensional cephalometric and photographic measures may not fully capture the three-dimensional nature of dentofacial relationships.

Future research should explore the interaction between vertical and sagittal skeletal patterns, incorporate three-dimensional imaging and dynamic smile analysis, and evaluate how these factors collectively influence the perception and stability of maxillary incisor inclination.

## 6. Conclusions

This study indicates that maxillary incisor inclination is generally preserved throughout orthodontic treatment in adults, even when vertical facial patterns differ. This suggests that clinicians effectively maintain incisor torque during treatment. The way in which the incisors relate to facial and growth axes, however, is not uniform. In normodivergent and hypodivergent individuals, these axes can serve as dependable guides during treatment finishing. In hyperdivergent patients, the skeletal features are less reliable, and final maxillary incisor position should instead be guided primarily by smile analysis and soft tissue balance. Recognizing these pattern tendencies may help clinicians refine decision-making and adapt finishing goals more precisely.

## Figures and Tables

**Table 1:** Comparison of cephalometric and dental parameters between T1 and T2 in normodivergent, hypodivergent, and hyperdivergent groups.

Groups	Mean ± SD			Mean ± SD			Mean ± SD		
	Normo T1 (*n* = 66) 20 ♂ & 46 ♀	Normo T2 (*n* = 66) 20 ♂ & 46 ♀	*p*	Hypo T1 (*n* = 35) 17 ♂ & 18 ♀	Hypo T2 (*n* = 35) 17 ♂ & 18 ♀	*p*	Hyper T1 (*n* = 43) 11 ♂ & 32 ♀	Hyper T2 (*n* = 43) 11 ♂ & 32 ♀	*p*
Age (year)	26.805 ± 11.585	32.102 ± 24.984	0.065	26.929 ± 9.344	29.824 ± 9.911	0.000	28.005 ± 9.381	30.900 ± 9.866	0.000
Maxillary incisors/NA	22.086 ± 9.635	22.109 ± 7.690	0.984	23.994 ± 9.181	26.326 ± 6.857	0.130	20.063 ± 7.790	20.495 ± 6.650	0.718
Maxillary incisors/PP	112.212 ± 9.563	112.423 ± 7.709	0.839	113.464 ± 10.254	116.077 ± 7.971	0.145	109.321 ± 8.177	109.614 ± 7.575	0.804
Maxillary incisors/SN	102.935 ± 15.655	104.921 ± 7.184	0.297	106.966 ± 10.211	109.457 ± 7.617	0.135	98.640 ± 9.221	96.058 ± 14.929	0.308
ANB	3.073 ± 2.940	3.003 ± 3.156	0.765	2.343 ± 2.419	2.646 ± 4.231	0.671	4.444 ± 2.184	4.512 ± 2.017	0.676
Facial axis/NBa	90.236 ± 3.822	90.788 ± 3.561	0.058	93.737 ± 4.766	93.520 ± 4.572	0.636	84.179 ± 4.670	83.886 ± 4.454	0.488
Facial axis/H	117.092 ± 3.506	118.213 ± 5.873	0.067	120.328 ± 4.370	120.851 ± 6.375	0.555	111.358 ± 4.969	109.460 ± 7.647	0.069
Growth axis/NBa	93.454 ± 3.452	93.466 ± 4.105	0.973	95.085 ± 6.027	94.971 ± 6.081	0.771	88.253 ± 4.818	87.669 ± 4.439	0.181
Growth axis/H	120.377 ± 3.064	120.755 ± 3.752	0.251	122.585 ± 3.852	122.526 ± 4.128	0.873	115.619 ± 5.287	115.533 ± 5.690	0.822
Maxillary incisors/NBa	85.857 ± 11.856	86.071 ± 9.497	0.829	89.208 ± 14.383	91.551 ± 12.003	0.141	80.267 ± 8.900	80.383 ± 7.672	0.926
Maxillary incisors/H	112.058 ± 9.360	111.980 ± 7.243	0.939	114.183 ± 10.581	116.449 ± 7.771	0.177	107.207 ± 8.982	106.265 ± 7.981	0.478
MP/SN	32.165 ± 2.464	32.365 ± 3.706	0.609	23.857 ± 4.306	25.297 ± 5.078	0.011	41.812 ± 3.771	41.258 ± 4.325	0.276
IMPA	94.917 ± 9.420	94.936 ± 9.509	0.983	96.014 ± 7.711	98.974 ± 8.861	0.014	93.502 ± 8.124	95.409 ± 7.751	0.064
Interincisal angle	128.350 ± 13.045	127.211 ± 10.659	0.403	131.380 ± 13.863	125.503 ± 9.752	0.061	126.902 ± 13.602	123.674 ± 10.403	0.074

**Table 2:** Means of selected clinical crown axis measurements at T1 and T2 in the 3 divergence groups.

Groups	Mean ± SD		*p*	Mean ± SD		*p*	Mean ± SD		*p*
	Normo T1 (*n* = 66) 20 ♂ & 46 ♀	Normo T2 (*n* = 66) 20 ♂ & 46 ♀		Hypo T1 (*n* = 35) 17 ♂ & 18 ♀	Hypo T2 (*n* = 35) 17 ♂ & 18 ♀		Hyper T1 (*n* = 43) 11 ♂ & 32 ♀	Hyper T2 (*n* = 43) 11 ♂ & 32 ♀	
CCA/V	−4.74 ± 9.124	−4.62 ± 6.841	0.902	−4.83 ± 7.610	−3.43 ± 7.397	0.219	−6.35 ± 9.365	−4.30 ± 8.484	0.194
CCA/Sn′	0.45 ± 8.862	0.06 ± 6.704	0.710	−0.69 ± 9.698	−0.31 ± 7.936	0.770	0.88 ± 9.008	2.67 ± 6.802	0.241
CCA/H	85.05 ± 9.008	85.35 ± 6.813	0.754	85.34 ± 7.604	86.57 ± 7.397	0.284	83.60 ± 8.862	85 ± 8.053	0.383

**Table 3:** Comparisons of T2 − 1 differences in selected cephalometric measurements between the 3 divergence groups.

Groups	Mean ± SD			*p*	Σ		
	Normo δ (*n* = 66) 20 ♂ & 46 ♀	Hypo δ (*n* = 35) 17 ♂ & 18 ♀	Hyper δ (*n* = 43) 17 ♂ & 18 ♀		Between Normo & Hypo	Between Normo & Hyper	Between Hypo & Hyper
Age (year)	2.459 ± 1.356	2.895 ± 1.923	2.895 ± 1.437	0.240			
Maxillary incisors/NA	0.0227 ± 8.914	2.331 ± 8.889	0.432 ± 7.808	0.427			
Maxillary incisors/PP	0.210 ± 8.406	2.431 ± 9.654	0.293 ± 7.687	0.419			
Maxillary incisors/SN	1.986 ± 15.354	2.491 ± 9.633	−2.581 ± 16.410	0.202 NS			
ANB	−0.697 ± 1.883	0.302 ± 4.184	0.067 ± 1.049	0.774			
Facial axis/NBa	0.551 ± 2.320	−0.217 ± 2.688	−0.293 ± 2.743	0.165			
Facial axis/H	1.121 ± 4.891	0.522 ± 5.185	−1.897 ± 6.671	0.020	1.000	0.019	0.172
Growth axis/NBa	0.012 ± 2.849	−0.114 ± 2.309	−0.583 ± 2.814	0.525			
Growth axis/H	0.377 ± 2.645	−0.060 ± 2.205	−0.086 ± 2.493	0.559			
Maxillary incisors/NBa	0.213 ± 8.006	2.342 ± 9.194	0.116 ± 8.215	0.413			
Maxillary incisors/H	−0.077 ± 8.223	2.265 ± 9.719	−0.941 ± 8.629	0.255			
MP/SN	0.200 ± 3.159	1.440 ± 3.155	−0.553 ± 3.289	0.025	0.197	0.694	0.021
IMPA	0.019 ± 7.512	2.960 ± 7.742	1.906 ± 6.580	0.113			
Interincisal angle	−1.139 ± 10.995	−5.877 ± 11.736	−3.227 ± 11.536	0.137			

SD: Standard deviation. NS: not statistically significant.

**Table 4:** Pearson Correlation Coefficients Between Clinical Crown Axis and Skeletal Axes at T1 and T2 in Different Divergence Groups.

		Hypodivergent *n* = 35		Normodivergent *n* = 66		Hyperdivergent *n* = 43	
		CCA/V	CCA/Snpg′	CCA/V	CCA/Snpg′	CCA/V	CCA/Snpg′
**Facial axis/NBa**	T1	0.44 **	0.27	0.37 **	0.15	0.02	−0.09
	T2	0.16	−0.01	0.21	−0.01	0.042	−0.11
**Facial axis/H**	T1	0.44 **	0.13	0.30 *	0.06	−0.06	−0.13
	T2	0.27	0.04	−0.05	−0.15	0.19	0.09
**G axis/NBa**	T1	−0.23	−0.27	0.25 *	0.04	0.05	−0.05
	T2	−0.34 *	−0.52 **	0.14	−0.09	0.09	0.45
**G axis/H**	T1	0.01	−0.26	0.24	0.01	−0.06	−0.11
	T2	−0.02	−0.34 *	−0.13	−0.27 *	−0.11	−0.19

** Correlation is significant at *p* < 0.001. * Correlation is significant at *p* < 0.05.

## Data Availability

All data generated or analyzed during this study are included in this published article.

## References

[1] Doshi P., Kalia A., Patil W., Gupta G., Ahmed D.I. (2017). Evaluation of the Effect of Maxillary Incisor Labiolingual Inclination & Antero-Posterior Position on Smiling Profile Esthetics—A Computer Aided Photographic Study. Sci. J. Res. Dent..

[2] Cao L., Zhang K., Bai D., Jing Y., Tian Y., Guo Y. (2011). Effect of maxillary incisor labiolingual inclination and anteroposterior position on smiling profile esthetics. Angle Orthod..

[3] Richmond S., Klufas M.L., Sywanyk M. (1998). Assessing incisor inclination: A non-invasive technique. Eur. J. Orthod..

[4] Ghahferokhi A.E., Elias L., Jonsson S., Rolfe B., Richmond S. (2002). Critical assessment of a device to measure incisor crown inclination. Am. J. Orthod. Dentofacial Orthop..

[5] Knösel M., Kubein-Meesenburg D., Sadat-Khonsari R. (2007). The third-order angle and the maxillary incisor’s inclination to the NA line. Angle Orthod..

[6] Sabri R. (2005). The eight components of a balanced smile. J. Clin. Orthod..

[7] Jacobson A. (2006). Radiographic Cephalometry from Basics to 3-D Imaging.

[8] Ellis E., McNamara J.A. (1986). Cephalometric evaluation of incisor position. Angle Orthod..

[9] Naini F.B. (2011). Facial Aesthetics: Concepts and Clinical Diagnosis.

[10] Arnett W., McLaughlin R. (2004). Facial and Dental Planning for Orthodontists and Oral Surgeons.

[11] Ricketts R.M. (1961). Cephalometric analysis and synthesis. Angle Orthod..

[12] Downs W.B. (1956). Analysis of the Dentofacial Profile. Angle Orthod..

[13] Andrews W.A. (2008). AP relationship of the maxillary central incisors to the forehead in adult white females. Angle Orthod..

[14] Ghaleb N., Bouserhal J., Bassil-Nassif N. (2011). Aesthetic evaluation of profile incisor inclination. Eur. J. Orthod..

[15] Albwardi M., Albwardi S., Dobaian K., Alqahtani K., Altayir A., Almutawa A. (2022). The Influence of Maxillary Incisor Labiolingual Inclination on Smiling Profile Esthetics Among Saudis. Cureus.

[16] Proffit W.R., Fields H.W., Larson B.E., Sarver D.M. (2019). Contemporary Orthodontics.

[17] Hussels W., Nanda R.S. (1984). Analysis of factors affecting angle ANB. Am. J. Orthod..

[18] Bishara S.E., Jakobsen J.R. (1998). Changes in overbite and face height from 5 to 45 years of age in normal subjects. Angle Orthod..

[19] Assi S.B., Salameh Z., Hanna A., Aybout J., Macari A. (2021). Orthodontic Treatment Effect on Inclination of Maxillary Incisors and Growth Axes in Adult Patients with Various Mandibular Divergent Patterns. J. Contemp. Dent. Pract..

[20] Naini F.B., Manouchehri S., Al-Bitar Z.B., Gill D.S., Garagiola U., Wertheim D. (2019). The maxillary incisor labial face tangent: Clinical evaluation of maxillary incisor inclination in profile smiling view and idealized aesthetics. Maxillofac. Plast. Reconstr. Surg..

[21] Cheng H.C., Cheng P.C. (2017). Factors affecting smile esthetics in adults with different types of anterior overjet malocclusion. Korean J. Orthod..

[22] Palmares S., Caseiro R., Pereira R., Jardim L. (2024). Perception of maxillary incisor inclination and its correlation with dental cephalometric measurements. J. Orthod..

[23] Flores-Mir C., Silva E., Barriga M.I., Lagravere M.O., Major P.W. (2004). Lay person’s perception of smile aesthetics in dental and facial views. J. Orthod..

[24] Sarver D.M., Ackerman M.B. (2003). Dynamic smile visualization and quantification: Part 1. Evolution of the concept and dynamic records for smile capture. Am. J. Orthod. Dentofacial Orthop..

[25] Burns N.R., Musich D.R., Martin C., Razmus T., Gunel E., Ngan P. (2010). Class III camouflage treatment: What are the limits?. Am. J. Orthod. Dentofacial Orthop..

[26] Troy B.A., Shanker S., Fields H.W., Vig K., Johnston W. (2009). Comparison of incisor inclination in patients with Class III malocclusion treated with orthognathic surgery or orthodontic camouflage. Am. J. Orthod. Dentofacial Orthop..

[27] Kau C.H., Bakos K., Lamani E. (2020). Quantifying changes in incisor inclination before and after orthodontic treatment in class I, II, and III malocclusions. J. World Fed. Orthod..

[28] Schudy F.F. (1964). Vertical growth versus anteroposterior growth as related to function and treatment. Angle Orthod..

[29] Enlow D.H., Hans M.G. (1996). Essentials of Facial Growth.

[30] Zierhut E.C., Joondeph D.R., Artun J., Little R.M. (2000). Long-term profile changes associated with successfully treated extraction and nonextraction Class II Division 1 malocclusions. Angle Orthod..

[31] Chirivella P., Singaraju G.S., Mandava P., Reddy V.K., Neravati J.K., George S.A. (2017). Comparison of the effect of labiolingual inclination and anteroposterior position of maxillary incisors on esthetic profile in three different facial patterns. J. Orthod. Sci..

[32] Sarver D.M. (2001). The importance of incisor positioning in the esthetic smile: The smile arc. Am. J. Orthod. Dentofacial Orthop..

[33] Ackerman M.B., Ackerman J.L. (2002). Smile analysis and design in the digital era. J. Clin. Orthod..

[34] Graber T.M., Vanarsdall R.L., Vig K.W. (2011). Orthodontics: Current Principles and Techniques.

